# Differential selection on pollen and pistil traits in relation to pollen competition in the context of a sexual conflict over timing of stigma receptivity

**DOI:** 10.1093/aobpla/plw061

**Published:** 2016-09-23

**Authors:** Åsa Lankinen, Maria Strandh

**Affiliations:** 1 Swedish University of Agricultural Sciences, Plant Protection Biology, PO Box 102, S-230 53 Alnarp, Sweden; 2 Department of Biology, Lund University, Ecology Building, S-223 62 Lund, Sweden

**Keywords:** *Collinsia heterophylla*, cryptic self-incompatibility, mixed mating, pollen competition, pollen deposition schedules, sexual conflict, sexual selection, timing of stigma receptivity

## Abstract

Sexual conflict and its evolutionary consequences are understudied in plants, but the theory of sexual conflict may help explain how selection generates and maintains variability. Here, we investigated selection on pollen and pistil traits when pollen arrives sequentially to partially receptive pistils in relation to pollen competition and a sexual conflict over timing of stigma receptivity in the mixed-mating annual *Collinsia heterophylla* (Plantaginaceae). In this species the conflict is generated by early fertilizing pollen that reduces seed production, which is counteracted by delaying receptivity in the recipient. We performed sequential two-donor pollinations at early floral developmental stages involving two pollen deposition schedules (with or without a time lag of 1 day), using only outcross or self and outcross pollen. We investigated pollen and pistil traits in relation to siring success (male fitness) and seed production (female fitness). In contrast to previous findings in receptive pistils in *C. heterophylla* and in other species, last arriving pollen donors showed highest siring success in partially receptive pistils. The last male advantage was weaker when self pollen was the first arriving donor. Two measures of germination rate (early and late) and pollen tube growth rate of first arriving donors were important for siring success in crosses with a time lag, while only late germination rate had an effect in contemporary crosses. Curiously, late stigma receptivity was negatively related to seed production in our contemporary crosses, which was opposite to expectation. Our results in combination with previous studies suggest that pollen and pistil traits in *C. heterophylla* are differentially advantageous depending on stage of floral development and varying pollen deposition schedules. Variation in success of these traits over floral development time may result from sexually antagonistic selection.

## Abstract (precis)

Sexual conflict and its evolutionary consequences are understudied in plants, but the theory of sexual conflict may help explain how selection generates and maintains variability in both plants and animals. Here, we show that pollen and pistil traits involved in a sexual conflict over timing of stigma receptivity are differentially advantageous during pollen competition depending on stage of floral development and varying pollen deposition schedules. Variation in success of these traits over floral development time may result from sexually antagonistic selection.

## Introduction

A main aim in biology is to understand the biological and genetic diversity within natural populations. Balancing selection, where the relative selective benefit of alleles differ depending on their context, is a common explanation for maintenance of variation, involving the mechanisms heterozygote advantage, negative frequency dependent selection, spatial or temporal environmental heterogeneity, antagonistic pleiotropy and sexual antagonism (reviewed by Delph and Kelly 2014). Sexual antagonism can result from sexual conflict between alleles at the same or different interacting loci (intra- vs. interlocus, Parker 1979; Rice and Chippindale 2001). In the latter case the outcome of divergent evolutionary interests of males and females are expressed in sexual selection to maximize fitness in either sex at the expense of the mating partner, which can lead to sexually antagonistic coevolution between male and female traits (Parker 1979; Arnqvist and Rowe 2005; Kokko and Jennions 2014). Sexually antagonistic coevolution is believed to generate mechanisms that augment genetic variation (Bonduriansky 2011; Gavrilets 2014). For example, frequency dependent selection can favour polymorphism of antagonistic defence traits that can escape the negative impact of an antagonistic trait of the mating partner (Gavrilets and Waxman 2002; Gavrilets and Hayashi 2005), which appears to occur in some insect species (Svensson *et al.* 2009; Green *et al.* 2014).

Sexual selection is a broad and active research area in animals (e.g., Eldakar *et al.* 2009; Martínez-Padilla *et al.* 2014; Green *et al.* 2014; van Lieshout *et al.* 2014; see review by Hosken and House 2011), while in plants the incidence and evolutionary consequences of sexual selection is much less studied (Moore and Pannell 2011; Lankinen and Karlsson Green 2015). In plants, sexual selection can involve competition for pollinators ((Delph and Ashman 2006; Cocucci *et al.* 2014) but is particularly likely to take place during pollen competition in the pistil (the equivalent of sperm competition, Bernasconi *et al.* 2004), favouring pollen traits that confer high competitive ability (e.g. pollen tube growth rate, Snow and Spira 1991) and pistil traits that enhance pollen competition to favour some pollen over others (e.g. a long style, Mulcahy 1983; Ramesha *et al.* 2011). Sexual conflict is poorly investigated in plants but some conflicts have been identified, for example over flower size (Delph *et al.* 2004, 2010), pollen production (Duffy and Johnson 2014), timing of stigma receptivity (Lankinen *et al.* 2006; Lankinen and Kiboi 2007; Madjidian *et al.* 2012a) and seed provisioning (Queller 1984; Willi 2013). To date, however, we have limited knowledge regarding whether sexually antagonistic selection can generate variability in plant traits involved in sexual conflict (cf. Gavrilets 2014).

One important difference between most animals and plants is that plants are sessile and mating occurs by proxy involving a pollinating agent (e.g. insects). Dependence on a pollinator means that plants are unable to fully control the mating process, such as how much, how often and which type of pollen arrives to the stigma (e.g., Spira *et al.* 1996; Karron *et al.* 2006; Burkhardt *et al.* 2009; Mitchell *et al.* 2013; Pannell and Labouche 2013). For this reason, sexual selection of pollen and pistil traits during pollen competition is strongly influenced by the random component of pollen deposition schedules, such that the first arriving pollen often will have a reproductive advantage (Marshall and Ellstrand 1985; Burkhardt *et al.* 2009). To understand selection of pollen and pistil traits it is therefore crucial to evaluate their relation to reproductive success across multiple pollen deposition schedules.

In *Collinsia heterophylla*, a mixed-mating (combining outcross and self pollination in the same individual) annual, we have found evidence for a sexual conflict over timing of stigma receptivity (Lankinen *et al.* 2006). Pollen with a capacity to advance receptivity can fertilize the ovules early thereby securing paternity which comes at a recipient cost of reduced seed set and seed biomass (Lankinen and Kiboi 2007; Madjidian *et al.* 2012a). Our previous studies also suggest that late onset of stigma receptivity can increase seed production in mixed donor pollinations, particularly at the earliest stage of floral development (Lankinen *et al.* 2016), indicating that this trait can counteract the negative influence of early-germinating pollen. Another previous study in *C. heterophylla* showed a first male advantage on receptive stigmas following sequential hand-pollination (Lankinen and Madjidian 2011). We do not know if this applies also to partially receptive stigmas, as may be expected in terms of the sexual conflict because of the benefit of early-arriving pollen to outcompete later arriving pollen (see Lankinen *et al.* 2006). Such first male advantage could potentially make it more difficult for recipients to control timing of fertilization, placing the pollen donor in “power” of the conflict (cf. Parker 1979; Kokko and Jennions 2014). Apart from hindering early germination of pollen (Lankinen *et al.* 2016), late stigma receptivity in *C. heterophylla* has been shown to be beneficial in terms of enhanced pollen competition in receptive pistils, leading to reduced inbreeding depression and increased offspring quantity and quality (Lankinen and Armbruster 2007; Madjidian 2011; Lankinen and Madjidian 2011). Given that late stigma receptivity appears adaptive it is puzzling that this trait is highly variable within natural populations, generally ranging between 1-2 to 4 days after flower opening (Lankinen *et al.* 2007, 2016). One hypothesis is that ineffective control of germination of early arriving pollen, e.g. when pollen from different donors arrive sequentially (see Burkhardt *et al.* 2009), selects for pistil acceptance of early fertilization to avoid costs of reduced seed set (cf. Lessells 2006). Variability in timing of pistil acceptance of pollen would also impact selection on pollen competitive traits, linking pollen and pistil traits within hermaphrodite individuals. For example, pollen with a capacity to advance germination could be expected to perform relatively better on pistils with late stigma receptivity (Hersh *et al.* 2015). To understand how sexually antagonistic selection potentially can generate variability in timing of stigma receptivity and pollen competitive traits it would be highly informative to learn about the impact of pollen deposition schedules on male siring success and seed production, as well as the relation to pollen and pistil traits.

The aim of this study on *C. heterophylla* was to investigate potential influence of the sexual conflict on variability in pollen and pistil traits by investigating pollen competition in partially receptive pistils when pollen arrives sequentially. We performed controlled two-donor pollinations at early floral developmental stages, involving either (i) donors applied consecutively at about the same time (no time lag) or (ii) donors applied with a time lag of 1 day. Siring success of competing donors was determined with genetic markers. We also estimated pollen and pistil traits of competing donors and recipients. Moreover, because we have previously seen that siring success of self pollen relative to outcross pollen was reduced in receptive but not in partially receptive pistils (Lankinen *et al.* 2016), suggesting cryptic self-incompatibility (Bateman 1956; Cruzan and Barrett 1993; Goodwillie *et al.* 2005) only at later stages of floral development, we also performed crosses where pollen donors competed with self pollen. To understand selection on pollen and pistil traits in relation to a sexual conflict in a mixed mating species it is crucial to combine studies on pollen deposition schedules with the influence of competing self pollen. We asked; (i) How is siring success (male reproductive success) in partially receptive pistils affected by deposition schedule of competing donors, cross type (outcross or self + outcross), and by pollen and pistil traits? (ii) How is seed production (female reproductive success) affected by deposition schedule of competing donors, cross type, and by timing of pistil receptivity in recipients? (iii) Are pollen and pistil traits correlated when measured within the same hermaphrodite individuals?

## Methods

### Plant material

*C.* *heterophylla* (Plantaginaceae) is a self-compatible, hermaphrodite annual native to California Floristic Province, North America (Newson 1929; Neese 1993) that can be found below 1000 m a.s.l. on dry slopes in meadow-like environments shaded by trees. It flowers between March and June depending on elevation and site conditions. Insect-pollination occurs by long-tongued, nectar-feeding bees (Armbruster *et al.* 2002), and population outcrossing rates range between 0.29 and 0.82 with microsatellite markers (Kalisz *et al.* 2012; Hersh *et al*. 2015).

The purple to white flowers are arranged in whorls on spikes with a zygomorphic corolla forming an upper and lower lip. Flowers contain one single-style pistil and four epipetalous stamens (Armbruster *et al.* 2002). When flowers open, the pistil is undeveloped with a short style and a non-receptive stigma, and the anthers are undehisced. During floral development, anthers dehisce at a rate of approximately one per day during 4 consecutive days, while the style elongates and the stigma matures and becomes receptive usually at day 2–3 after flower opening (Lankinen e*t al.* 2007). Self pollination occurs at a late stage, when the style is sufficiently long to come into contact with the dehisced anthers (Kalisz *et al.* 1999; Armbruster *et al.* 2002). Flowers develop into seed capsules that contain up to 20 seeds (Madjidian and Lankinen 2009). In this study we refer to floral developmental stage 1–4 as day 1–4 after flower opening which roughly corresponds to number of dehisced anthers (following previous studies, see Armbruster *et al.* 2002; Lankinen *et al.* 2007). Stage 0 = day of flower opening. Field experiments have confirmed that pollinators visit flower at all developmental stages and that this can generate seeds as early as 1 day after flower opening, i.e. stage 1 (Hersh *et al.* 2015). Thus, we can expect expression of the sexual conflict under natural conditions.

Plants used in this study originated from a natural population in California, Mariposa county (situated at N 37.50196; W 120.12360), sampled in 2008 by collecting seeds from over 200 maternal open-pollinated individuals. Previous to the experiment plants were grown for one generation in the greenhouse to establish an outbred base population. Plants were raised from cold-stratified seeds and grown in pollinator-proof conditions during winter and early spring of 2013.

### Sequential two-donor pollinations

**Figure 1:**
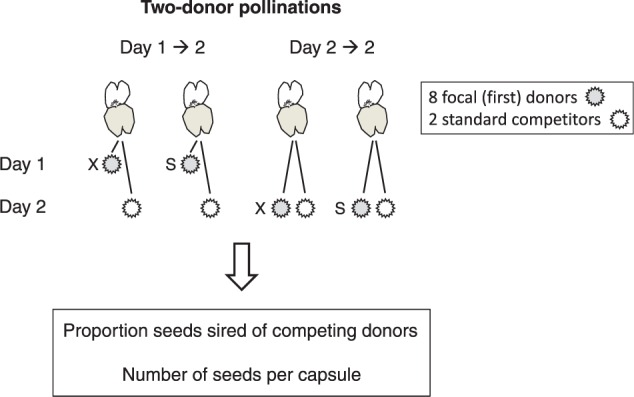
Experimental design of sequential two-donor pollinations involving a focal pollen donor applied previous to a standard competitor at early floral development in *C. heterophylla*. The focal donor was applied on unrelated (X) or self (S) pistils, while the standard donor was always unrelated to the recipient. In crosses performed with a time lag of 1 day, ‘Day 1→2’, the first donor was added on day 1 after flower opening and the standard donor was added on the next day (day 2). In crosses without a time lag, ‘Day 2→2’, the first donor was applied to the stigma a few minutes before the standard donor on day 2 after flower opening. Seed capsules resulting from the crosses were evaluated with respect to proportion seeds sired by the competing donors (male reproductive success) and number of seeds per capsule (female reproductive success).

To investigate effects of pollen deposition schedule at early floral developmental stages we performed two-donor hand-pollinations with sequential deposition of two pollen donors (Fig. 1). Flowers emasculated at flower opening were crossed twice either (i) on day 1 and day 2 after flower opening, i.e. with a time lag of 1 day (pollen deposition schedule Day 1→2) or (ii) repeatedly on day 2, a few min between crosses (pollen deposition schedule Day 2→2). All emasculations and hand-pollinations were conducted at approximately the same time every day. Pollen from each donor was added to microscopic slides. We transferred pollen from the slides directly to the stigma by gently dipping stigmas in the pile of pollen until it was covered in pollen. We aimed to add approximately the same amount of pollen in all pollinations.

In total, we used eight focal pollen donors that were pollinated first, two standard donors that were pollinated last and nine recipients (Fig. 1). We used only two standard donors to reduce variability contributed by the second donor. Our design made it possible to compare the relationship between trait values for focal donors and for recipients. Each recipient was hand-pollinated with two focal donors and with self pollen that all competed with one of the two standard donors, i.e. every recipient had the same standard donor. All recipient—donor combinations were repeated four times per pollen deposition schedule. Number of crosses per recipient = (2 + 1) focal donors × 2 pollen deposition schedules × 4 replicates = 24. Individual pollen donors were used at least on two unrelated recipients (involving different standard donors) and on self pistils. Crosses were distributed evenly in time with respect to treatments and focal donor identity. To be able to compare both donor and recipient effects in relation to outcross or self + outcoss pollination, and also how pollen and pistil traits correlated in the same hermaphrodite plants, most focal donors and recipients represented the same hermaphrodite individual. There is thus some dependence between focal donors and recipients, but as male performance and female performance of trait values were determined over a different set of mates we can consider these estimates as independent.

Ripe seed capsules were collected. We determined number of seeds per capsule and stored seeds in a refrigerator for later determination of paternity shares.

### Determination of paternity shares

Leaves from parent individuals used in crossings were collected and dried 24 h in silica gel. DNA was extracted from two leaves per individual using ZR Plant/Seed DNA MiniPrep-kit (Zymo Research) according to the manufacturer’s instructions. For the offspring generation, twenty seeds from each crossing method (with or without a time lag) and crossing type (outcross or self + outcross) were used for paternity analyses (randomly chosen from a pool of seeds of the four replicates). The seeds were kept in petri dishes with moist (tap water) filter paper overnight to allow cutting of seeds before DNA-extraction. DNA was extracted from half the seed in 50 µl Quick Extract Seed DNA Extraction Solution (Epicentre) according to the manufacturer's protocol. Four polymorphic microsatellite loci (A11, A106, A116 and C1) developed for *C. sparsiflora* (JW Wright and ML Stanton, USDA, Forest Service Pacific Southwest Research Station, unpubl. data) were amplified in one multiplex PCR reaction (containing four primer pairs) per individual (parent or seed), with fluorophore-labelled (FAM for C1 and A11; HEX for A116 and A106) forward primers and non-labelled reverse primers. Each PCR reaction contained 1 µl DNA extract (∼5–10 ng DNA), 0.2 µM of each primer, 1× Multiplex PCR Master Mix and 0.6 µl Q-Solution from the Qiagen Multiplex PCR Kit (Qiagen) in a total volume of 10 µl. The following cycling parameters were used: 95 °C for 15 min and then 35 cycles of 94 °C for 30 s, 56 °C for 90 s and 72 °C for 60 s. A final extension step at 60 °C for 10 min was applied. The PCR products were analysed by GeneScan fragment analysis, adding GeneScan 500 ROX Size Standard (Applied Biosystems) to the samples, on an ABI3730XL DNA Analyzer instrument (Applied Biosystems) at Uppsala Genome Center, Sweden. The resulting chromatograms were used for genotyping by size determination with the Microsatellite Plugin in the software Geneious v6.1.4 (Biomatters Ltd.). Paternity of each seed from the two possible donors was determined by careful inspection of chromatograms. Data was kept for further analyses only for crossings where paternity genotypes could unambiguously be separated from each other. In total, we used data from 46 out of 66 donor-donor-recipient combinations.

### Estimates of pollen and pistil traits

We estimated two measures of germination rate, pollen tube growth rate and pollen and pistil-based onset of timing of stigma receptivity in all plants involved in the current study (*n* = 13), allowing us not only to connect siring success and seed production to these traits but also to test for correlations between traits in the same hermaphrodite individual.

Germination rate after 15 min and after 1 h 45 min (hereafter denoted ‘early germination rate’ and ‘germination rate’), and pollen tube growth rate were measured *in vitro* by germination in Hoekstra medium (Hoekstra and Bruinsma 1975). Pollen taken from two flowers per individual plant was sprinkled onto a drop of medium and incubated for (i) 15 min (early germination rate) and (ii) 1h 45 min (germination rate and pollen tube growth rate) in a dark chamber at a constant temperature of 20–21 °C (see also Lankinen *et al.* 2009). Both measures of pollen germination rate were estimated as the percentage pollen germination in a sample by classifying the first 100 pollen grains encountered in a randomly chosen area as either germinated or non-germinated. Pollen tube growth rate was assessed by measuring the length of 10 pollen tubes per sample in a randomly chosen area under a standard light microscope and taking the average. *In vitro* pollen tube growth rate has previously been shown to correlate positively with *in vivo* pollen tube growth rate (Lankinen *et al.* 2009).

Pollen- and pistil-based onset of stigma receptivity were estimated in a separate crossing experiment performed on recipients (*n* *=* 11) that were full-siblings to the nine recipients per focal pollen donors and the two standard donors used in the sequential two-donor crosses. Pollen-based onset of stigma receptivity refers to the earliest floral stage during which pollen from a given donor resulted in seed set on other individuals (data averaged over two to three recipients), indicating pollen influence on timing of stigma receptivity. Likewise, pistil-based onset of stigma receptivity refers to the earliest floral stage at which flowers on a given recipient set seed with pollen from other individuals (averaged over three donors) (Madjidian *et al.* 2012b; Hersh *et al.* 2015; Lankinen *et al.* 2016). Pollen source was the same donors as in the main crosses (focal and standard) and for evaluation of other pollen traits (*n* *=* 13). One-donor pollinations were conducted on emasculated flowers at each of four floral developmental stages (1–4 days after flower opening) using the method described above. Emasculations (at flower opening) and crosses were performed at about the same time every day. Four hours after hand-pollination, half of the style was removed to allow fertilization only when the stigma and upper part of the pistil were receptive to pollen (Lankinen and Kiboi 2007). Each recipient was crossed with three unrelated donors and replicated twice per stage, thus number of crosses per recipient = 3 donors × 4 stages × 2 replicates = 24. Pollen donors were used evenly among recipients, i.e. on two to three recipients. Pollen- and pistil-based onset of stigma receptivity were calculated as the average stage when seeds first were formed in the crosses involving a given donor or a given recipient (Madjidian *et al.* 2012b; Hersh *et al.* 2015; Lankinen *et al.* 2016).

### Statistics

To analyze effects of pollen deposition schedule and type of cross on the relative proportion seeds sired by the first vs. the standard donor over all recipient and donor combinations we performed logistic regression in R (R Development Core Team 2015), i.e. a generalized linear model with a binomial distribution and a logit link function. The model included pollen deposition schedule, cross type and their interaction. We controlled for overdispersion by refitting the model with quasibinomial errors. Statistical significance (*P* < 0.05) was assessed by testing the change in deviance between successive models with an *F*-test. All non-significant factors or interactions were excluded using backward deletion of higher-order interactions. In an additional analysis we used the proportion seeds sired (siring success) by first arriving donor, allowing us to test treatment effects of the fixed factors pollen deposition schedule, cross type, their interaction, standard donor and the random factor recipient nested within standard donor (a nested ANOVA in SPSS 2014). We included standard donor rather than focal donor because this resulted in a more balanced design. A non-significant interaction (*P* > 0.10) was removed from the final model. Analysis of number of seeds per capsule (averaged over the up to four replicate crosses per unique recipient and donor combination) was conducted with a similar nested ANOVA.

Relationships between dependent traits (siring success of the first pollen donor and number of seeds per capsule) and pollen/pistil traits of donors/recipients were investigated by a Linear regression analyses involving several values of y for each value of x (Sokal and Rohlf 1995). We performed these analyses on (i) all data in order to investigate the relationships over all treatments as well as (ii) separate analyses for each of the two pollen deposition schedules (pooling outcross + outcross and self + outcross). We also conducted Linear regression for each of the four treatment groups. Because standard donor differed in siring success (see ‘Results’ section), we used the focal donor—standard donor combination as independent data points of siring success (averaged over recipients) per treatment group and correlated these values with the difference in pollen traits between focal and standard donor. For regressions involving pistil-based onset on receptivity we used recipient means of the dependent variables per treatment group. It should be noted that we cannot separate effects of donors and recipients in these analyses, but using mean values made the data more balanced.

Type III sum of squares were used in ANOVAs. All proportions were arcsine transformed.

## Results

### Siring success as an effect of pollen deposition schedule and type of cross

**Figure 2:**
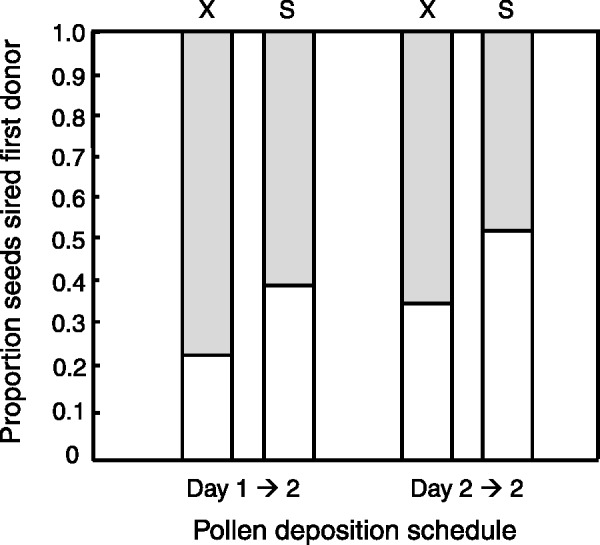
Proportion seeds sired by first arriving pollen donor (white) following sequential two-donor crosses at early floral development where the first donor is competing with a standard donor (gray) on unrelated (X) or self (S) pistils in crosses with a time lag (Day 1→2) or without a time lag (Day 2→2).

In our two-donor crosses at early developmental floral stages both pollen deposition schedule (with or without a time lag) and type of cross (outcross + outcross or self + outcross) influenced proportion seeds sired of the first applied donor relative to the standard donor applied last (Logistic regression; Pollen schedule: *F*_1,43_ = 5.23, *P* = 0.027, Cross type: *F*_1,43_ = 8.94, *P* = 0.0046), while the pollen schedule by cross type-interaction was non-significant (*P* = 0.87). In most cases the donor applied last (standard) had higher siring success than the first donor (mean per treatment and cross type ranged between 47.3 and 77.6 %, Fig. 2). The superiority of the second donor was particularly evident for crosses with a time lag, i.e. when the first donor arrived to the stigma 1 day prior to the second donor. Early arriving self pollen performed relatively better than early arriving outcross pollen for both crosses with and without a time lag (Fig. 2).

An analysis including standard donor and recipient plant nested within standard donor showed the same pattern as the logistic regression with significant effects of pollen deposition schedule and cross type on siring success of first donor (Table 1), and no interaction effect (*P* = 0.70) (Fig. 2). The two standard donors, but not recipients, affected siring success differently (Table 1, mean ± SE: donor 63: 0.727± 0.033, *n* = 22; donor 136: 0.578 ± 0.051, *n* = 22).

**Table 1.** Nested ANOVA for proportion seeds sired of first arriving pollen donor when competing with a standard donor in sequential two-donor crosses with or without a time lag at early floral development, or number of seeds produced per capsule following these crosses (average of up to four crosses).

	Proportion seeds sired first donor			Number of seeds per capsule		
Source of variation	df	*F*	*P*	df	*F*	*P*
Pollen deposition schedule	**1,32**	**7.82**	**0.009**	1,32	0.795	0.38
Cross type	**1,32**	**5.01**	**0.032**	1,32	2.41	0.13
Standard donor	**1,7.93**	**5.82**	**0.043**	1,7.98	0.036	0.85
Recipient (St donor)	8,32	1.62	0.16	**8,32**	**5.24**	**< 0.001**
Pollen schedule × type	–	**-**	**-**	–	**-**	**-**

First arriving donor was either competing on unrelated or self pistils (=cross type), and applied one day ahead or immediately before the standard outcross donor (pollen deposition schedule). Recipients are nested within standard donor. Significant values are indicated in bold.

### Relation between siring success of early arriving donors and pollen and pistil traits

The coefficient of variation (cv) for pollen traits of the eight focal (first applied) donors and the two standard competing donors ranged between 10 and 32 % (mean ± SD, cv; early germination rate: 0.380 ± 0.073, 19.1 %, germination rate: 0.496 ± 0.157, 31.7 %, pollen tube growth rate: 0.279 ± 0.081 mm, 105 min^−^^1^, 29.0 %, pollen-based onset of receptivity: 2.78 ± 0.29 days after flower opening, 10.4 %). Pistil-based onset of receptivity of the nine recipient plants had a cv of 24.5 % (2.60 ± 0.64 days after flower opening).

**Figure 3:**
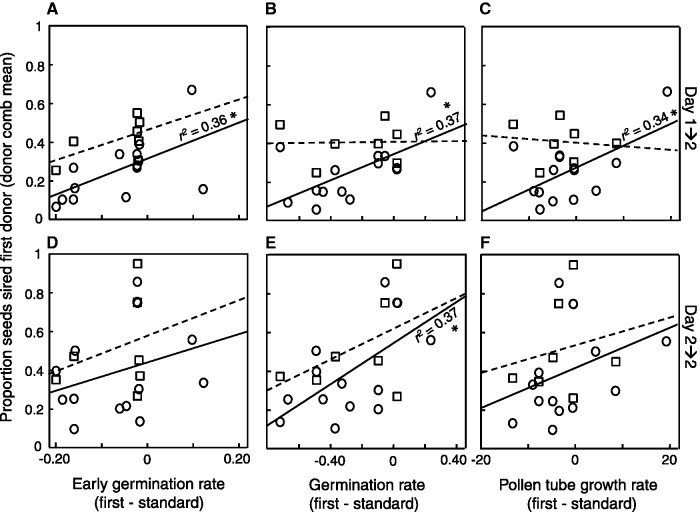
Relationship between proportion seeds sired by first arriving pollen donor when competing with a standard donor (averaged for each first (focal) and standard donor combination) and difference in pollen traits between focal and standard donor (evaluated in germination medium). Sequential two-donor pollinations where performed at early floral development (A–C) with a time lag of one day (Day 1→2) or (D-F) without a time lag (Day 2→2). Circles and solid lines = outcross pollen, squares and dashed lines = self pollen of first donor. *r^2^*-values are shown for significant (*P* < 0.05, *) relationships (tested separately for the four experimental groups).

**Figure 4:**
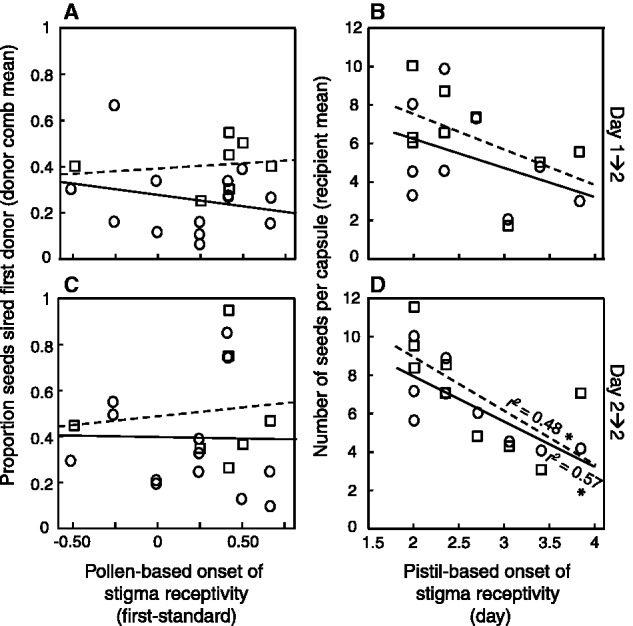
(A,C) Relationship between proportion seeds sired by first arriving pollen donor when competing with a standard donor (averaged for each first (focal) and standard donor combination) and difference in pollen-based onset of stigma receptivity between focal and standard donor. (B,D) Relationship between number of seeds per capsule averaged for each recipient and pistil-based onset of stigma receptivity. Sequential two-donor pollinations where performed at early floral development (A,B) with a time lag of 1 day (Day 1→2) or (C,D) without a time lag (Day 2→2). Circles and solid lines = outcross pollen, squares and dashed lines = self pollen of first donor. *r^2^*-values are shown for significant (*P* < 0.05, *) relationships (tested separately for the four experimental groups). Pollen- and pistil-based onset of stigma receptivity were evaluated in separate crosses.

Male siring success of focal (first applied) donor (averaged for each focal and standard donor combination) was significantly positively correlated only with the difference in germination rate between focal and standard donor over all crosses with and without a time lag (Table 2, Fig. 3 and 4A and B, *y* = 0.432 + 0.389*x*). Separate analyses of the two pollen deposition schedules (pooling outcross + outcross and self + outcross treatments) showed that germination rate was strongly related to siring success in crosses without a time lag (*y* = 0.356 + 0.831*x*) but not in crosses with a time lag (Table 2). The opposite result was seen for differences in early germination rate, i.e. this trait was important for siring success in crosses with a time lag (Table 2, Fig. 3A and D, *y* = 0.521 + 0.536*x*). Differences in pollen tube growth rate showed a positive relationship with siring success for crosses with a time lag in unrelated (outcross) pistils (Linear Regression; *F*_1,12_ = 5.38, *P* = 0.039, *y* = 0.271 + 0.011*x*, Fig. 3C), but no significant correlations was seen in the other treatment groups (Table 2, Fig. 3C and F). Differences in pollen-based onset of stigma was unrelated to siring success, with the trend that early onset was linked with high siring success in crosses with a time lag (Table 2, Fig. 4A and B).

**Table 2.** Linear regression analyses for proportion seeds sired of first arriving pollen donor (averaged for each first (focal) and standard donor combination) in sequential two-donor crosses with or without a time lag at early floral development in relation to difference in pollen traits between first and standard donor.

								Pollen-based	
		Early germination rate		Germination raten		Pollen tube growth		onset of receptivity	
		(first-standard)		(first-standard)		(first-standard)		(first-standard)	
Source of variation	df	*F*	*P*	*F*	*P*	*F*	*P*	*F*	*P*
Pollen deposition schedule day 1→2 + day 2→2									
Donor combination	13,28	2.02	0.058	2.02	0.058	2.02	0.058	2.02	0.058
Regressed pollen trait	1,12	3.61	0.082	**7.72**	**0.017**	2.12	0.17	0.001	0.99
Deviation from regression	12,28	1.68	0.13	1.33	0.26	1.86	0.086	2.19	0.043
Pollen deposition schedule day 1→2									
Donor combination	13,7	1.63	0.26	1.63	0.26	1.63	0.26	1.63	0.26
Regressed pollen trait	1,12	**6.01**	**0.031**	2.97	0.11	2.06	0.18	0.090	0.77
Deviation from regression	12,7	1.18	0.43	1.41	0.33	1.51	0.30	1.75	0.023
Pollen deposition schedule day 2→2									
Donor combination	13,7	2.57	0.11	2.57	0.11	2.57	0.11	2.57	0.11
Regressed pollen trait	1,12	1.15	0.30	**5.94**	**0.031**	1.10	0.31	0.033	0.86
Deviation from regression	12,7	2.54	0.11	1.86	0.21	2.55	0.11	2.78	0.091

First donor was applied 1 day ahead or immediately before the standard outcross donor (pollen deposition schedule day 1→2 vs. pollen deposition schedule day 2→2). Significant values of regressed traits are indicated in bold. Data for outcross + outcross and self + outcross pollinations were pooled. Because the same donor combination was used for each of the four traits, test results are identical for donor combination.

Pistil-based onset of receptivity was unrelated to siring success (averaged over recipients) (Linear Regression; Recipient: *F*_7,20_ = 0.828, *P* = 0.58, Pistil-based onset of receptivity: *F*_1,6_ = 0.411, *P* = 0.55, Deviation from regression: *F*_6,20_ = _ _0.903, *P* = 0.51).

### Seed production as an effect of pollen deposition schedule and cross type

The probability that a cross was successful was 80.2 % (206 out of 257) and did not differ between the four treatment groups (χ^2^ = 2.42, df = 3, *P* = 0.49).

Number of seeds per capsule (averaged over the up to four replicate crosses per recipient and focal-standard donor combination) showed a strong influence of recipient plant nested within standard donor, but no effects of standard donor, pollen deposition schedule or cross type (Table 1) or the pollen schedule by cross type interaction (*P* = 0.30). The non-significant trend was that fewer seeds were produced in crosses with a time lag and outcross pollen (mean ± SE: day 1→2, outcross + outcross: 5.85 ± 0.84, *n* = 11, day 2→2, outcross + outcross: 6.75 ± 0.63, *n* = 11, day 1→2, self + outcross: 6.74 ± 0.72, *n* = 11, day 2→2, self + outcross: 7.08 ± 0.78, *n* = 11).

### Seed production in relation to pistil-based onset of receptivity

Number of seeds produced (averaged over recipients) was significantly negatively related to pistil-based onset of receptivity over all four treatment groups (Table 3, Fig. 4, *y* = 0.142 − 65.8*x*). Separate analyses for crosses with and without a time lag showed that late stigma receptivity was negatively correlated with reduced seed set only in crosses without a time lag (pooled for outcross + outcross and self + outcross) (Table 3, Fig. 4B, *y* = -2.24 – 22.2*x*).

**Table 3.** Linear regression analyses for number of seeds per capsule (averaged for each recipient) following sequential two-donor crosses with or without a time lag at early floral development in relation to pistil-based onset of receptivity.

Source of variation	df	*F*	*P*
Pollen deposition schedule day 1→2 + day 2→2			
Recipient	8,27	3.20	0.011
Pistil-based onset of receptivity	1,7	**19.9**	**0.0029**
Deviation from regression	7,27	1.29	0.29
Pollen deposition schedule day 1→2			
Recipient	8,9	1.59	0.25
Pistil-based onset of receptivity	1,7	3.59	0.10
Deviation from regression	7,9	1.20	0.39
Pollen deposition schedule day 2→2			
Recipient	8,9	2.50	0.097
Pistil-based onset of receptivity	1,7	**18.0**	**0.0038**
Deviation from regression	7,9	0.801	0.61

First pollen donor was applied 1 day ahead or immediately before a standard donor (pollen deposition schedule day 1→2 vs. pollen deposition schedule day 2→2). Significant values are indicated in bold. Data for outcross + outcross and self + outcross pollinations were pooled.

## Discussion

Sequential two-donor crosses during early floral development in the mixed mating *C.* *heterophylla* revealed that pollen arriving 1 day ahead of its competitor had lower relative siring success. The same applied to early arriving self pollen, but self pollen performed relatively better than outcross pollen as the first donor. Both measures of germination rate (early and late) and pollen tube growth rate of first arriving donors were positively correlated with siring success in crosses with a time lag, but only late germination rate had an effect in crosses without a time lag. Contrary to expectation, late stigma receptivity was linked to reduced seed set in crosses without a time lag, indicating that early receptivity can be beneficial in some instances.

### Selection on pollen traits following sequential crosses at early stages

It has commonly been observed that early arriving pollen has a reproductive advantage over later arriving competitors (Marshall and Ellstrand 1985; Spira *et al.* 1996; Jolivet and Bernasconi 2007). Crosses in receptive pistils of *C. heterophylla* also showed this pattern (Lankinen and Madjidian 2011). This led us to hypothesize that pollen arriving early to partially receptive stigmas would have higher siring success. Contrary to expectation, we found that the later arriving pollen donor performed better. This was the case both in crosses with a time lag of 1 day and in crosses without a time lag, but the time lag decreased the success of early arriving pollen. In should be noted that we only used two last pollen donors in all crosses. It is conceivable that our result was influenced by the competitive ability of these two donors rather than the order of arrival of the donors. However, we judge this possibility unlikely because the two standard donors differed in siring success, and the results were consistent over competition with eight first pollen donors and self pollen. We have currently no knowledge regarding what is happening in the pistil. We can hypothesize that the last male advantage is at least partly a result of that pollen germination and/or tube growth is much slower at stage 1 than stage 2. Another possible explanation is that pollen grains reaching the ovules at the earliest stage are harming ovules by too early penetration (cf. ovule usurping by self pollen, Waser and Price 1991). The former hypothesis is less likely as seeds can be formed at stage 1 when half the style is cut off 4 h after pollination.

In terms of the sexual conflict over timing of stigma receptivity (Lankinen and Kiboi 2007; Madjidian *et al.* 2012a) we would expect that the capacity of rapid germination at the earliest stage would give a reproductive advantage over later arriving pollen in crosses with a time lag (Lankinen *et al.* 2006). On the other hand, because pollen has no control over when to arrive to the stigma, the ability to perform well at an early stage should be selected for if this ability is higher than the performance of other pollen at an early stage (in other flowers). Indeed, we found that early germination rate, germination rate and pollen tube growth rate correlated positively with siring success of the first arriving (outcross) pollen donor in crosses with a time tag, implying that pollen donors differ in their ability to fertilize the ovules at the earliest stage. Studies in other species (on receptive pistils) have shown that pollen tube growth rate often affect siring success (Snow and Spira 1991; Pasonen *et al.* 1999) but germination rate (Jolivet and Bernasconi 2007) and pollen size (McCallum and Chang 2016) can also have an impact. In mixed-donor pollinations performed at floral developmental stage 1–4 in *C. heterophylla*, late pollen-based onset of stigma receptivity was more important than pollen tube growth rate for siring success (Lankinen *et al.* 2016). In this study, pollen-based onset of stigma receptivity had no significant influence on siring success but the trend for crosses with a time lag was that early pollen-based onset was important. These results imply that pollen deposition schedule could matter for which pollen trait confers high reproductive success. On the other hand, this difference can be due to experimental variation. However, within the current experiment we found that only one pollen trait—pollen germination rate estimated after 1 h, 45 min—was important for siring success in crosses without a time lag, confirming variability in pollen performance depending on pollen deposition schedule. The fact that more pollen traits were important for siring success when there was a time period between pollinations could also have been attributed to floral developmental stage as the first donor was deposited at stage 1, i.e. on more unripe pistils. We do not know, however, why being deposited immediately before pollen from a second donor is disadvantageous. We can hypothesize that it is more difficult to germinate fast on a partially receptive stigmatic surface without previous pollen deposition. It is known from other species that pollen germination increases with pollen load size (cf. pollen population effect, Brewbaker and Majumder 1961; Cruzan 1986; Thomson 1989).

When early arriving pollen was applied to self pistils, the negative effect of arriving 1 day ahead of the competitor was reduced compared with on outcross pistils. This difference was noticeable also in crosses without a time lag. These results are in agreement with our previous studies suggesting that self pollen germinates more rapidly at early developmental stages in *C. heterophylla* (Lankinen and Kiboi 2007; Madjidian *et al.* 2012a) and that cryptic self-incompatibility is only present in receptive pistils (Lankinen *et al.* 2016). For self pollen, pollen traits appeared to be of less importance for siring success compared with outcross pollen. However, this result can be an effect of lower sample size. Pooling the data for both outcross + outcross and self + outcross pollinations showed that only early germination rate was important in crosses with a time lag, while only germination rate had an effect in crosses without a time lag. It is, however, clear from our results that we should presume that early arrival of self pollen to the stigma will increase self pollination more than expected, which would particularly occur in cases when geitonogamous pollination is common. Interestingly, our previous mixed-donor crosses indicated that late pollen-based onset of stigma receptivity in outcross pollen increased siring success when competing with self pollen (Lankinen *et al.* 2016). It would have been appealing to explore if this trait also could influence outcross siring success in sequential crosses, but this was beyond the scope of this study.

### Selection on timing of stigma receptivity following sequential crosses at early stages

Although it has been commonly assumed that enhanced pollen competition will improve offspring quality (e.g. seed traits) because deleterious alleles expressed during pollen tube growth can be purged (Mulcahy 1971, 1979; Walsh and Charlesworth 1992), the expectation of sexual conflict is instead a female fitness cost, e.g. expressed as negative effects on maternal seed traits (Parker 1979). Indeed, previous crosses in *C. heterophylla* involving two pollinations added sequentially with a time lag of 1 day showed that seed biomass was reduced compared with when only one pollination was added on the second day (Madjidian *et al.* 2012a). This effect was particularly pronounced in the comparison between crosses performed at stage 1 and 2 after flower opening or only at stage 2. Because a low pollen load size in the first pollination rather increased seed biomass than decreased it, pollen limitation is an unlikely explanation for this result. In this study—where sample size was smaller—we were unable to detect a significant difference between the two pollen deposition schedules but the trend was, as expected, lower number of seeds produced in the crosses with a time lag.

Given that seed set is impaired at early fertilization in *C. heterophylla* (Madjidian *et al.* 2012a) the last-male advantage detected in the current study indicates that pistils at least partly control timing of fertilization. In the previous mixed two-donor pollinations we found that recipients with late stigma receptivity produced more seeds, particularly at stage 1 (Lankinen *et al.* 2016). Curiously, there was no benefit of this trait at stage 2 (but rather a negative trend) but at later stages the relationship appeared positive again. These results may suggest that late stigma receptivity can mitigate the negative effect of early germinating pollen at stage 1, acting as a female antagonistic “defence” trait (Arnqvist and Rowe 2005), but for some unknown reason this trait is no longer effective at stage 2. In the current study we found a negative correlation between seed set and pistil-based onset of stigma receptivity in both outcross + outcross and self + outcross crosses without a time lag (performed at stage 2). A similar but non-significant negative relationship was seen for crosses with a time lag (performed at stage 1 and 2). Thus, we could not show a benefit of this pistil trait for seed set for either of our pollen deposition schedules, and for crosses without a time lag it instead appeared advantageous to become receptive early, presumably accepting early germinating pollen. In our experiment we cannot separate between the stage-specific effect and the effect of the time lag of pollen deposition and we do not know when fertilization occurs. One hypothesis is that pollen arriving at stage 2 has the upper hand in the sexual conflict (Parker 1979; Kokko and Jennions 2014) so that pollen is relatively better at germinating fast compared with the pistil capacity of hindering early germination. Moreover, pistil-based onset of stigma receptivity had no influence on siring success of the two competitors. This is in line with our previous mixed-donor crosses (Lankinen *et al.* 2016), and suggests that this trait is not used by the recipient to directly control fertilization but rather creating an arena for enhanced pollen competition.

We found no significant correlations between pollen and pistil traits. This is in contrast to a recent study that showed a negative correlation between pollen- and pistil-based onset of stigma receptivity across multiple populations (Hersh *et al.* 2015), potentially suggesting that the pollen ability to fertilize early is linked to the pistil ability to withstand early fertilization. However, it should be noted that the sample size in the current study was considerably lower.

### Sources of variability in pollen and pistil traits

Variability in pollen competitive ability has been proposed to persist due to mutation and recombination (Walsh and Charlesworth 1992), negative genetic correlations between sporophytic and gametophytic life stages (Walsh and Charlesworth 1992; Delph *et al.* 1997), genotype by environment interactions (Gillespie and Turelli 1989; Delph *et al.* 1997), frequency-dependent selection (Lankinen and Skogsmyr 2001) or lowered selection on pollen competitive ability in mixed-mating species (Mazer *et al.* 2010). It is also possible that sexually antagonistic selection can explain variability (Gavrilets 2014). For example, in spiders size-related male mating strategies were suggested to generate extreme male size variation (Neumann and Schneider 2015) and in diving beetles two polymorphic dorsal structures in females, with the function to reduce male mating attempts, were augmented by diversifying selection (Green *et al.* 2014). Sexually antagonistic selection in combination with variation in pollen arrival to the stigma may have generated the differential reproductive success seen for pollen traits and pistil-based timing of stigma receptivity in this and previous studies of *C. heterophylla* (Lankinen *et al.* 2016), and could at least partly have contributed to some of the variation found in pollen traits (Lankinen *et al.* 2009) and in timing of stigma receptivity (Lankinen *et al.* 2007, 2016).

We do not know which pollen deposition schedules are most commonly occurring under natural conditions. A field study confirmed that pollinators visit *C. heterophylla* flowers as early as stage 1 and that this can lead to seed production (Hersh *et al.* 2015). Field observations have also shown that flowers can be visited multiple times (Å. Lankinen, Swedish University of Agricultural Sciences and M.A. Madjidan, Lund University, unpubl. data). Because it is known that pollen carryover is frequent in insect-pollinated species (Morris *et al.* 1994; Ohashi and Thomson 2009) it is probable that pollen arrive in mixtures from different donors (Mitchell *et al.* 2013) but maybe not in even mixtures or equal proportions. In two-donor pollinations in violets, a pollen donor with higher pollen tube growth rate than the competitor sired most of the seeds already when present in low proportions of the pollen load (Lankinen and Skogsmyr 2002), indicating that presence of inferior pollen had a limited effect on the outcome of pollen competition. In the future, additional field studies would be valuable as well as studies exploring a variety of pollen deposition schedules under controlled conditions.

## Conclusions

In conclusion, sequential hand-pollinations at early floral stages in *Collinsia heterophylla* showed that pollen arriving first to the partially receptive stigma sired less of the seeds compared with a second arriving pollen donor, suggesting a second male advantage. This is in contrast to the first male advantage seen in receptive pistils of *C. heterophylla* (Lankinen and Madjidian 2011) as well as in other species (Marshall and Ellstrand 1985; Spira *et al.* 1996; Jolivet and Bernasconi 2007). A time lag of 1 day between pollinations reduced siring success of the first donor even more, particularly on unrelated pistils. Pollen traits were differently linked to siring success in the two investigated pollen deposition schedules, which could influence variability in these traits. Likewise, stigma receptivity was negatively correlated to seed production in crosses without a time lag (performed in stage 2), which was the opposite of the previously found positive relationship between these two traits in mixed donor pollinations in stage 1 (Lankinen *et al.* 2016). Potentially, late stigma receptivity can only mitigate costs of early-fertilizing pollen under certain circumstances. It is possible that the variability in pollen and pistil traits is a consequence of sexually antagonistic selection in this species (cf. Gavrilets 2014). Future studies should consider sexually antagonistic selection as an additional mechanism of balancing selection in plants that can maintain variation (Delph and Kelly 2014).

## Sources of Funding

This work was supported by the Carl Trygger Foundation, the Crafoord Foundation, the Swedish Research Council (to Å.L.) and by the Gösta and Anna-Birgit Henriksson Foundation (to M.S.).

## Contributions by the Authors

Å.L designed the study, M.S. designed and performed the molecular paternity screening, Å.L. analysed the data and wrote the article with help from M.S.

## Conflicts of Interest Statement

No conflicts of interest.
